# Sternocleidomastoid and Pectoralis Major Myositis Due to Streptococcus dysgalactiae Bacteremia in a Healthy Adult: A Rare Case

**DOI:** 10.7759/cureus.102883

**Published:** 2026-02-03

**Authors:** Usamah Al-Anbagi, Abdulqadir J Nashwan, Hatem A Abdulmajeed, Godwin J Wilson, Mohamed G Mohamedali

**Affiliations:** 1 Department of Internal Medicine, Hamad Medical Corporation, Doha, QAT; 2 Department of Nursing and Midwifery Research, Hamad Medical Corporation, Doha, QAT; 3 Department of Clinical Imaging, Hamad Medical Corporation, Doha, QAT; 4 Department of Laboratory Medicine and Pathology, Hamad Medical Corporation, Doha, QAT

**Keywords:** bacteremia, infectious myositis, magnetic resonance imaging, myositis, streptococcus dysgalactiae

## Abstract

*Streptococcus dysgalactiae* is a β-hemolytic group C/G *Streptococcus* increasingly recognized as a cause of invasive human disease. Although commonly associated with cellulitis, bacteremia, septic arthritis, and endocarditis, primary infectious myositis due to this organism is rare, particularly in immunocompetent adults. We report a case of a 33-year-old previously healthy male with a history of unexplained recurrent *Streptococcus dysgalactiae* bacteremia who presented with a five-day history of progressive right-sided neck and upper chest pain accompanied by low-grade fever. Magnetic resonance imaging demonstrated inflammatory changes involving the sternocleidomastoid and pectoralis major muscles without evidence of abscess formation or necrotizing infection. Blood cultures grew *Streptococcus dysgalactiae*, confirming infectious myositis secondary to bacteremia. The patient was treated with intravenous β-lactam therapy, followed by oral antibiotics, completing a total six-week course, with rapid clinical improvement and complete recovery. This case adds to the limited literature on *S. dysgalactiae*-associated myositis and underscores the importance of considering deep muscle involvement in patients presenting with focal musculoskeletal pain and concurrent group C/G streptococcal bacteremia. Early imaging and timely antimicrobial therapy are essential to prevent progression to severe or necrotizing disease.

## Introduction

*Streptococcus dysgalactiae* is a large-colony, β-hemolytic *Streptococcus* that reacts with Lancefield group C or G antisera and commonly colonizes the upper respiratory, gastrointestinal, and genitourinary tracts [1]. Although historically regarded as a low-virulence organism, advances in microbiologic and genomic characterization have demonstrated that *S. dysgalactiae* possesses multiple virulence determinants similar to those of *Streptococcus pyogenes*, including M proteins, streptolysins, streptokinase, and pyrogenic exotoxins [2]. These shared mechanisms explain its increasing recognition as an invasive human pathogen.

Clinically, *S. dysgalactiae* is associated with a broad spectrum of disease, most commonly cellulitis, pharyngitis, bacteremia, septic arthritis, and endocarditis. However, increasingly severe manifestations have been reported, including toxic shock-like syndrome, necrotizing soft-tissue infection, vertebral osteomyelitis, ocular involvement, and multiorgan dysfunction [3-7]. Recent epidemiological studies have shown a rising global incidence of invasive group C/G streptococcal infections, with rates in some regions approaching those of group A and group B streptococci [8-10].

Muscle involvement is an uncommon manifestation of *S. dysgalactiae* infection. Published reports describe presentations ranging from localized inflammatory myositis to rapidly progressive necrotizing myositis with fatal outcomes [7,8]. Because early symptoms may mimic autoimmune, neurologic, or mechanical musculoskeletal disorders, diagnosis can be challenging and delayed. Most reported cases involve the lower extremities or paraspinal musculature, while involvement of cervical or anterior chest wall muscles is exceedingly rare.

Here, we describe a rare case of concurrent sternocleidomastoid and pectoralis major myositis secondary to *S. dysgalactiae *bacteremia in a previously healthy adult. This report adds to the limited contemporary literature on invasive *S. dysgalactiae* infection and highlights the importance of early imaging and prompt antimicrobial therapy in patients presenting with focal musculoskeletal pain and concurrent bacteremia.

## Case presentation

History

A 33-year-old previously healthy male with no chronic medical conditions or prior surgeries presented with a five-day history of progressive right shoulder and anterior chest pain. The pain began gradually, was dull and persistent, and worsened with shoulder movement. There was no history of trauma, overuse, swelling, or skin changes before symptom onset. He also reported intermittent low-grade fever, malaise, and mild shivering, without rigors or night sweats. Two weeks earlier, he had experienced a short, self-limited febrile illness associated with vomiting that resolved spontaneously after two days. He denied respiratory symptoms, including cough, hemoptysis, or pleuritic chest pain, and reported no other joint pain, rash, or restricted movement elsewhere.

He had no constitutional symptoms, such as weight loss, anorexia, or night sweats, and reported no known tuberculosis exposure. There were no urinary or gastrointestinal complaints. Of particular relevance, he had a history of recurrent episodes of *Streptococcus dysgalactiae* bacteremia over the preceding months. Extensive investigations, including transesophageal echocardiography (TEE), positron emission tomography (PET), and multiple urine and stool cultures, had failed to identify an underlying infectious focus.

He reported no recent travel or sick contacts. He was an active smoker and occasional alcohol consumer for approximately 10 years. He denied intravenous drug use, tattooing, blood transfusion, or high-risk sexual activity. His family history was unremarkable for chronic or infectious diseases.

Examination

On examination, the patient was alert and oriented, with no acute distress. He was afebrile and appeared well hydrated. Vital signs were stable, with a temperature of 36.8°C, heart rate of 78 bpm, respiratory rate of 20 breaths/min, blood pressure of 125/78 mmHg, and oxygen saturation of 99% on room air. He was neither pale nor jaundiced, and there was no lymphadenopathy.

Local examination revealed tenderness and mild soft-tissue swelling over the right sternoclavicular region, extending superiorly toward the sternocleidomastoid and inferiorly toward the pectoralis major. The overlying skin was normal, without warmth, erythema, fluctuance, or deformity. There was no sinus tract, and the clavicle was non-tender. Right shoulder movement was limited by pain; active abduction reached 90°, and passive abduction reached 120°. Internal and external rotations were mildly restricted but painless at rest. No crepitus or sternoclavicular joint effusion was detected. Examination of other joints was unremarkable. The spine and sacroiliac joints were non-tender with normal alignment.

Cardiovascular assessment revealed a regular pulse and normal heart sounds. Respiratory examination demonstrated clear bilateral breath sounds with no adventitious sounds. The abdomen was soft, non-tender, and without organomegaly. Neurological examination, including cranial nerves, tone, power, and sensation, was entirely normal.

Investigations, management, and outcome

Two sets of blood cultures were obtained before antibiotics were initiated. Laboratory evaluation revealed leukocytosis (WBC = 18.6 × 10⁹/L), elevated C-reactive protein (191 mg/L), and an erythrocyte sedimentation rate (ESR) of 58 mm/hr. Liver enzymes were mildly elevated. Renal function, electrolytes, and urinalysis were within normal limits. Procalcitonin was slightly elevated. A summary of laboratory findings is provided in Table 1.

**Table 1:** Laboratory results. CRP: C-reactive protein; ALT: alanine aminotransferase; AST: aspartate aminotransferase; HbA1c: glycosylated hemoglobin; PT: prothrombin time; INR: international normalized ratio; APTT: activated partial thromboplastin time; ANA: antinuclear antibody; anti-dsDNA: anti-double stranded DNA; anti-RO52: anti-RO52 antibody; anti-SS-A: anti-Sjogren’s syndrome A antibody; anti-nucleosomes: anti-nucleosome antibody; anti-SM: anti-Smith antibody; anti-RNP: anti-ribonucleoprotein antibody; anti-histones: anti-histone antibody; anti-PCNA: anti-proliferating cell nuclear antigen antibody; anti-SS-B: anti-Sjogren’s syndrome B antibody; anti-ribosomal-P protein: anti-ribosomal P protein antibody; anti-JO1: anti-histidyl tRNA synthetase antibody; anti-AMA-M2: anti-mitochondrial M2 antibody; anti-centromere B: anti-centromere B antibody; anti-PM-Scl: anti-polymyositis scleroderma antibody; anti-CCP: anti-cyclic citrullinated peptide.

Parameters	On admission	On discharge	Reference values
Total leukocytes	20.6	12.6	(6.2 x10^3/uL)
Hemoglobin (gm/dL)	12.7	12	(13-17 gm/dL)
Hematocrit	37.3	34.9	(40-50%)
Platelet (x10^3/uL)	412	535	(150-410 x10^3/uL)
CRP mg/L	191	38	(0-5 mg/L)
Procalcitonin (ng/mL)	0.58	Not done	(<0.05 ng/mL)
Serum urea (mmol/L)	5.4	6	(2.5-7.8)
Serum creatinine (umol/L)	63	67	(62-106)
Serum sodium (mmol/L)	138	138	(133-146)
Serum potassium K (mmol/L)	4.1	4.3	(3.5-5.3)
Serum calcium (mmol/L)	2.4	Not done	(2.2-2.6)
Serum magnesium (mmol/L)	0.86	0.86	(0.7-1 mmol/L)
Serum total protein (gm/L)	85	71	(60-80)
Serum albumin (gm/L)	43	39	(35-50)
Alkaline phosphatase (U/L)	127	160	(40-129)
ALT (IU/L)	58	140	(0-41)
AST (IU/L)	39	66	(0-41)
Serum total bilirubin (mg/dl)	26	11	(0-21)
HbA1c	4.4	Not done	(<6%)
PT (seconds)	13.4	12.3	(9.4-12.5 seconds)
INR	1.2	1.1	<1
APTT (seconds)	32.7	33.8	(25.1- 36.5 seconds)
Rheumatoid factor	15	Not done	(0-15 IU/mL)
Anti-CCP	<8	Not done	(0-17 IU/mL)
ANA profile (includes anti-dsDNA, anti-RO52, anti-SS-A, anti-nucleosomes, anti-Sm, anti-RNP, anti-histones, anti-PCNA, anti-SS-B, anti-ribosomal-P-protein, anti-JO1, anti-AMA-M2, anti-centromere B, anti-PM-Scl antibodies)	Negative	Not done	Negative

**Figure 1:**
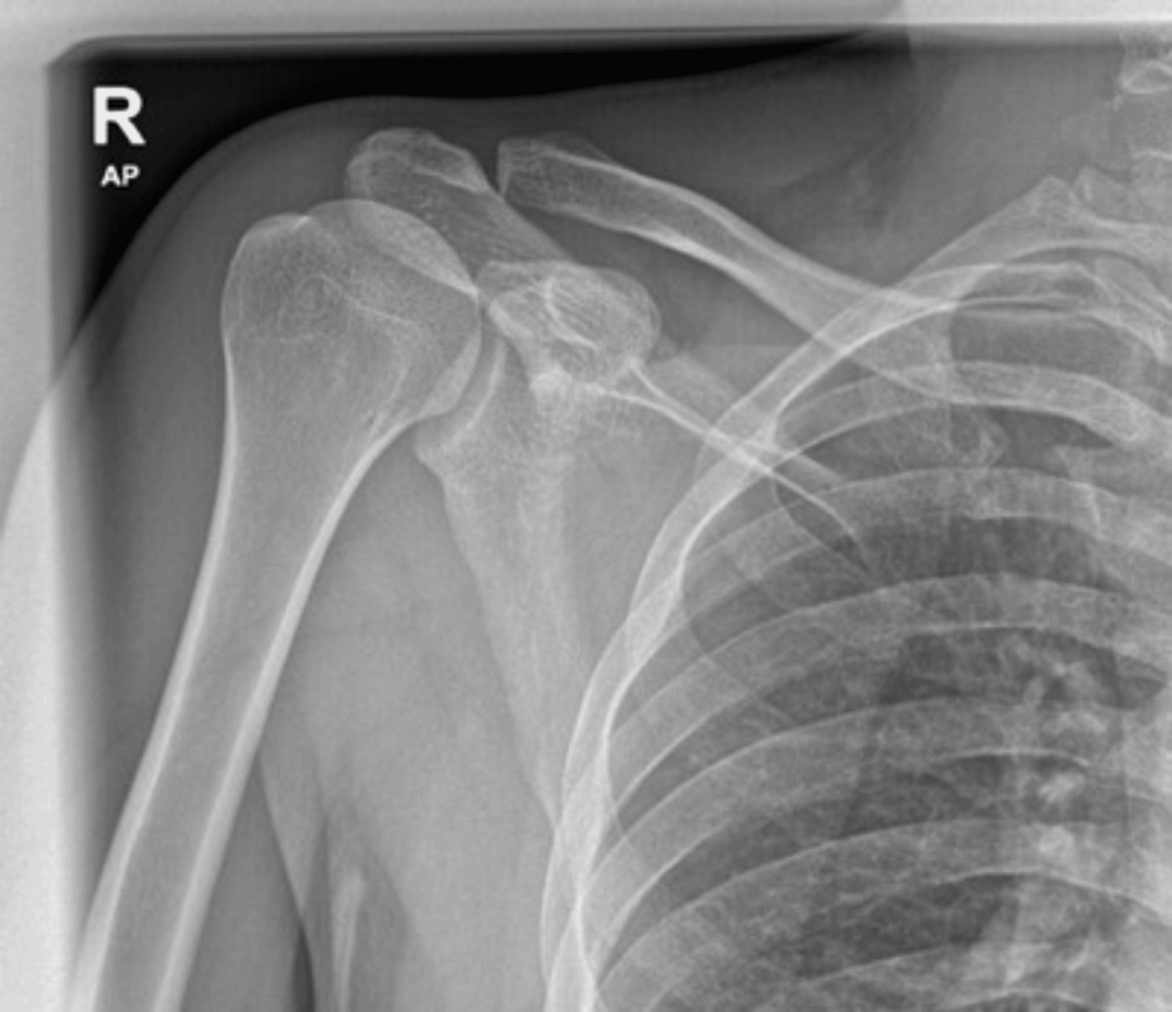
Right shoulder X-ray revealed unremarkable findings.

A shoulder X-ray (Figure 1) and ultrasound showed no abnormalities, with preserved joint spaces and no effusion, synovitis, or abscess formation. Due to persistent focal pain and localized tenderness over the sternoclavicular region, a contrast-enhanced magnetic resonance imaging (MRI) scan was performed on hospital day two.

**Figure 2:**
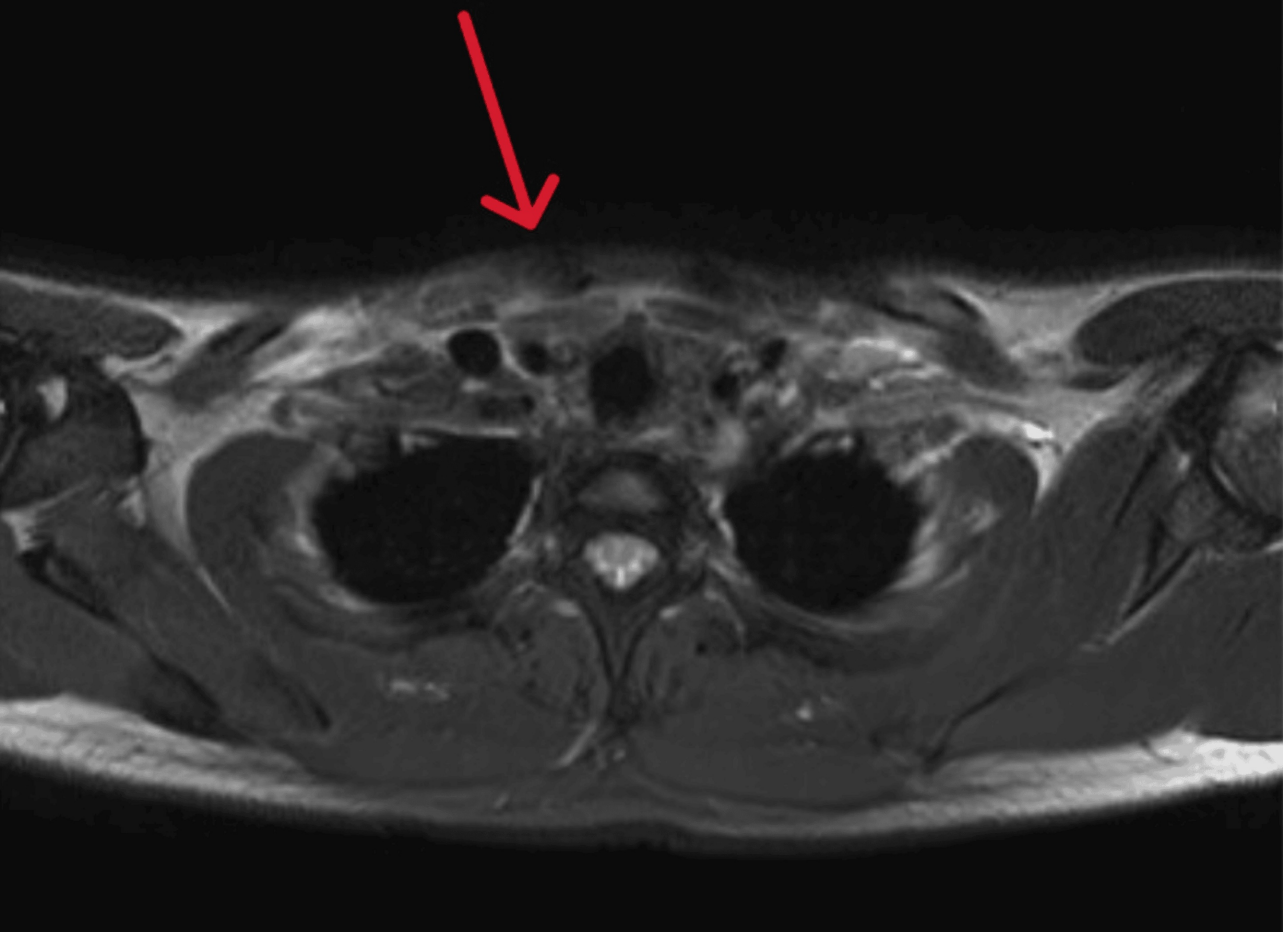
MRI of the chest (axial view) revealed soft tissue edema surrounding the proximal part of the clavicular head of the right sternocleidomastoid muscle (red arrow).

**Figure 3:**
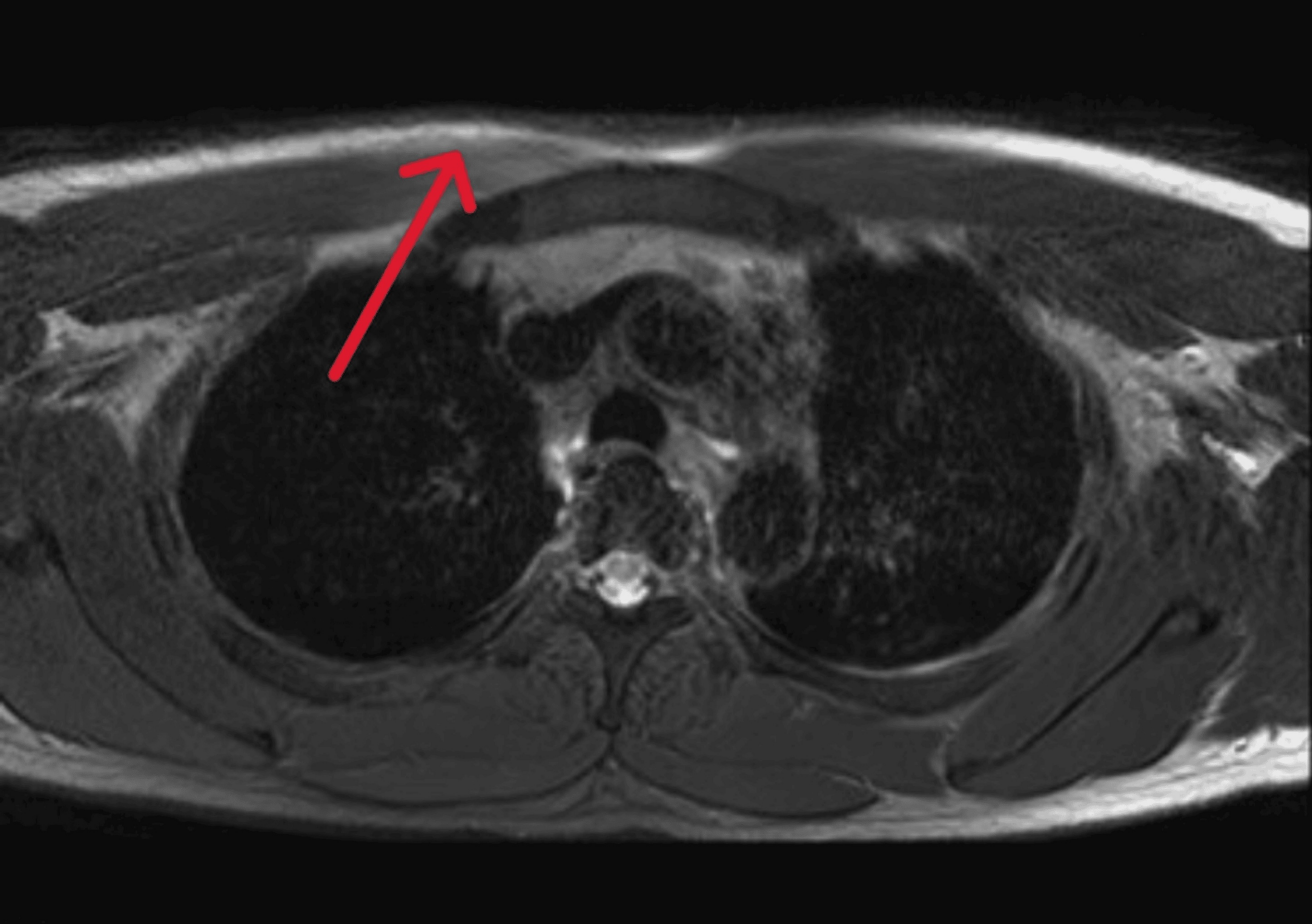
Axial short tau inversion recovery (STIR) MRI revealed deep soft tissue edema in the right proximal pectoralis major muscle (red arrow).

**Figure 4:**
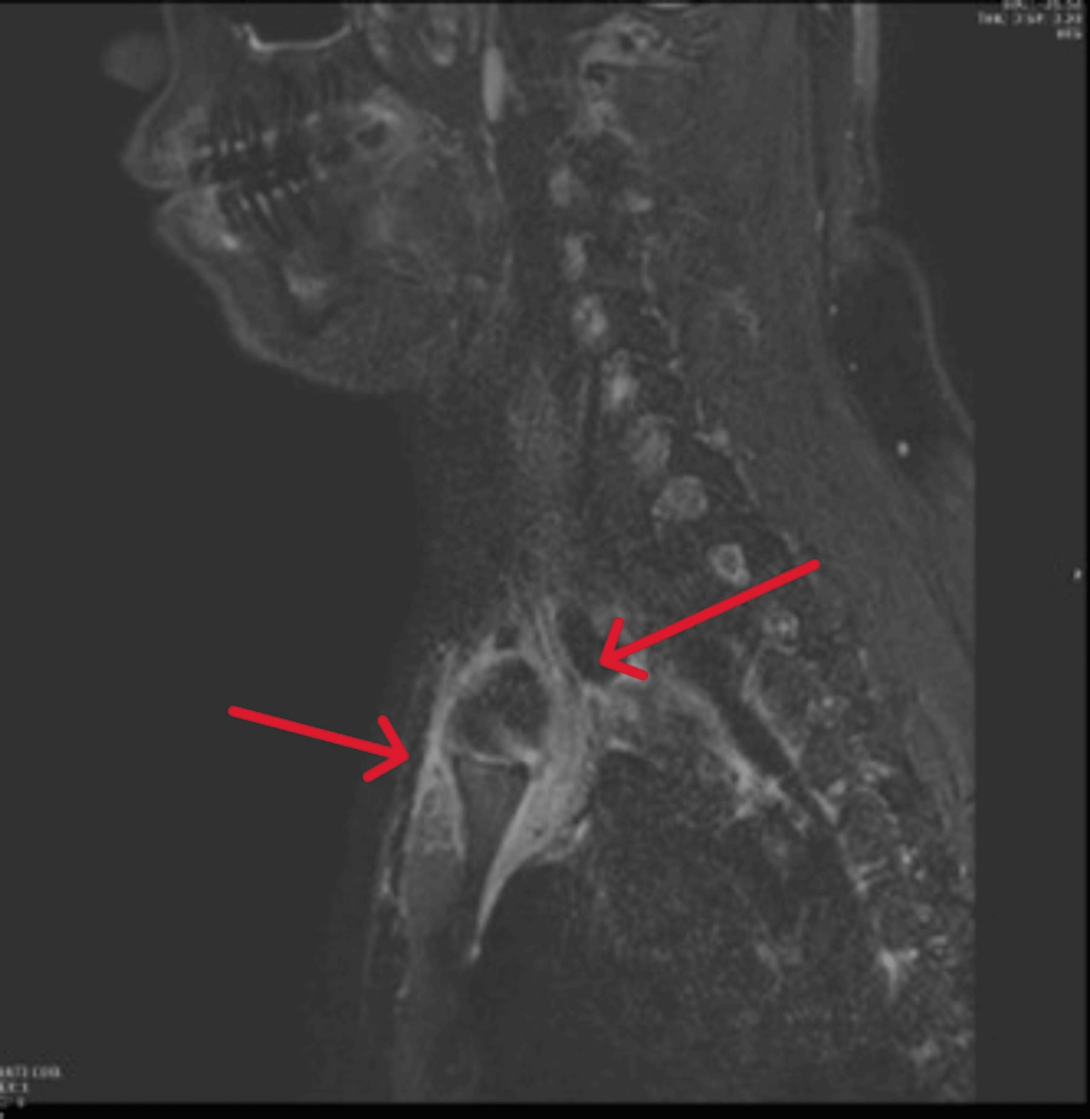
Sagittal short tau inversion recovery (STIR) MRI revealed deep soft tissue edema surrounding the sternoclavicular joints and the presternal and retrosternal soft tissues (red arrows).

**Figure 5:**
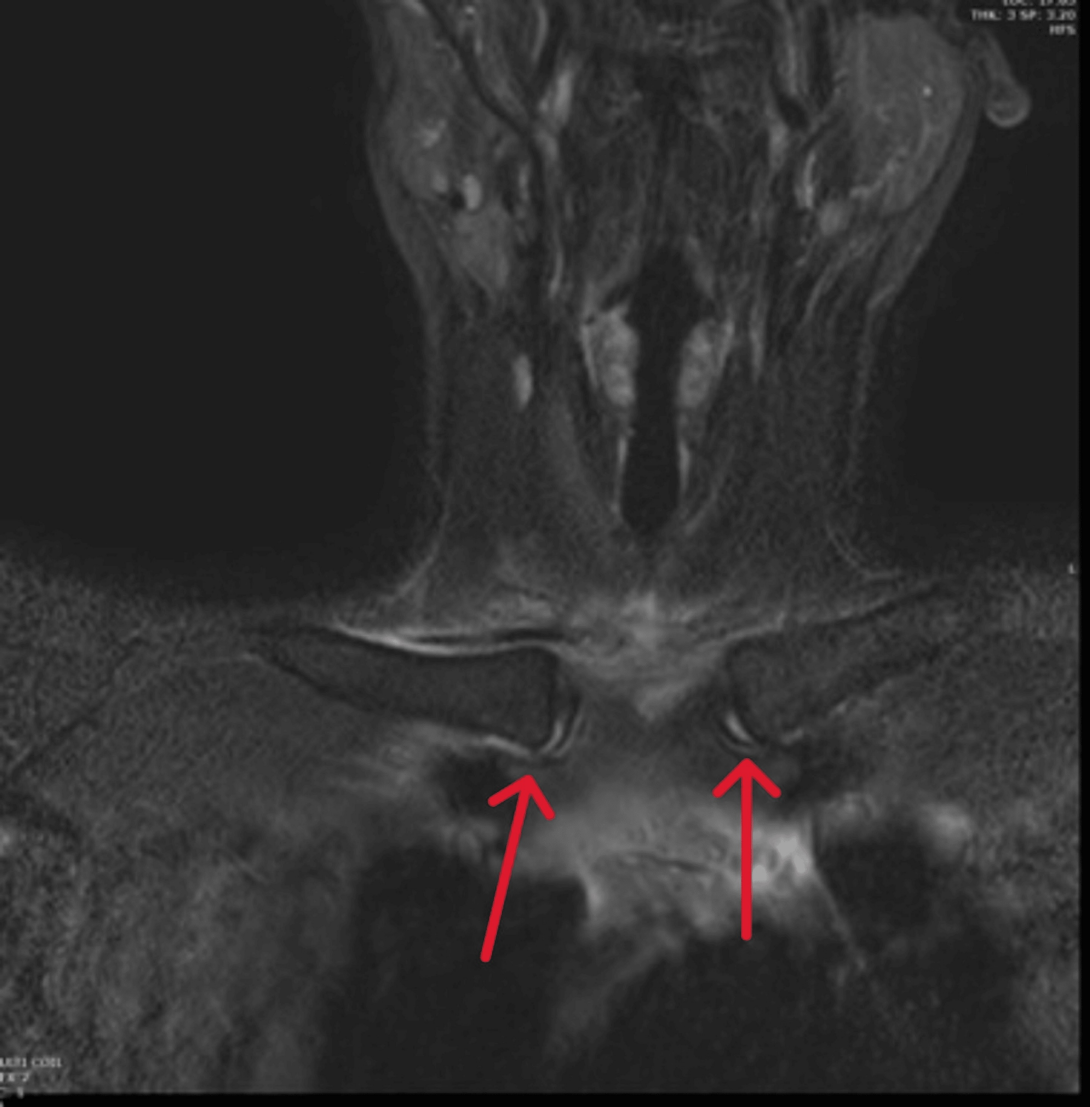
Coronal short tau inversion recovery (STIR) MRI revealed a fluid signal within both sternoclavicular joints (red arrows).

MRI demonstrated reactive edema and minimal joint effusion of the sternoclavicular joints without features of septic arthritis, erosions, or joint destruction (Figures 2-5).

On hospital day three, Blood cultures were processed using standard automated systems. The isolate was identified as *Streptococcus dysgalactiae* by matrix-assisted laser desorption/ionization time-of-flight mass spectrometry (MALDI-TOF MS; Bruker Daltonics, Billerica, MA), with a high-confidence score of 2.29. Antimicrobial susceptibility testing was performed according to the Clinical and Laboratory Standards Institute (CLSI) guidelines. The organism was susceptible to penicillin and ceftriaxone but resistant to clindamycin. Inducible clindamycin resistance was confirmed by a positive D-test. Repeat blood cultures obtained 72 hours after starting therapy were sterile. The patient was initially started on intravenous ceftriaxone 2 g daily. Following infectious disease consultation and review of susceptibility results, treatment was optimized to intravenous amoxicillin-clavulanate 1.2 g every eight hours for five days, then transitioned to oral amoxicillin-clavulanate 1 g twice daily to complete a six-week antimicrobial course.

Further evaluation, including chest radiography, urine and stool cultures, and transthoracic echocardiography to exclude endocarditis, revealed no additional infectious source. The patient’s symptoms improved significantly within several days, with resolution of fever, decreasing pain, and improved shoulder mobility. Inflammatory markers gradually normalized. He was discharged on hospital day seven in stable condition with instructions to continue oral therapy and attend follow-up. At the six-week review, he had almost complete recovery with only minimal residual discomfort.

## Discussion

*Streptococcus dysgalactiae* is increasingly recognized as an important cause of invasive human disease. Although once regarded as a relatively benign organism, genomic studies have demonstrated that it shares multiple virulence factors with *Streptococcus pyogenes*, including antiphagocytic M proteins, streptolysins, streptokinase, and pyrogenic exotoxins [1,2]. These attributes underpin its capacity to cause a broad spectrum of clinical syndromes, ranging from uncomplicated cellulitis to fulminant systemic infection [3-7]. Recent epidemiological data further indicate a rising global incidence of group C/G streptococcal disease, with rates in some regions approaching those of group A and group B streptococci [9,10].

The expanding clinical profile of *S. dysgalactiae* includes septic shock, multiorgan dysfunction, and deep soft-tissue infection [6,11]. Reported complications are diverse and include ocular involvement, necrotizing fasciitis, vertebral osteomyelitis, septic arthritis, and toxic shock-like syndromes, reflecting its invasive potential [4,5,12]. Although infectious myositis is well documented with other streptococcal species, it remains distinctly uncommon with *S. dysgalactiae*. Most described cases involve lower extremities or paraspinal musculature, and some initially resemble autoimmune or inflammatory myopathies, as observed in cases presenting with progressive limb weakness mimicking polymyositis [8].

Muscle involvement may range from localized inflammatory myositis to rapidly progressive necrotizing infection, with fatal outcomes reported [7]. Importantly, severe presentations can occur even in immunocompetent individuals. Case reports describe cervical osteomyelitis with atlantoaxial instability and panophthalmitis secondary to bacteremia, underscoring the need for vigilance when evaluating patients with focal pain and positive blood cultures [5,12].

In our patient, the affected anatomical region, encompassing the sternoclavicular area, sternocleidomastoid, and pectoralis major, can closely mimic sternoclavicular septic arthritis, deep neck infection, or early osteomyelitis. These conditions share overlapping symptoms, including localized pain, swelling, and restricted movement. Cross-sectional imaging is therefore critical for accurate differentiation. MRI remains the gold standard for assessing soft-tissue infection, enabling precise characterization of muscle inflammation and distinguishing it from abscess, necrotizing changes, and joint or bone pathology [13]. In this case, MRI findings confirmed isolated myositis without evidence of septic arthritis or destructive joint pathology.

Concurrent myositis of the sternocleidomastoid and pectoralis major muscles appears exceedingly rare, with no comparable cases identified in the literature. This unusual distribution increases diagnostic complexity, as it may mimic deep neck space infection, cervical lymphadenitis, or shoulder septic arthritis. The absence of necrotizing features or abscess formation allowed our patient to be managed successfully with antibiotic therapy alone. The likely pathogenesis in this case is hematogenous seeding during bacteremia, a mechanism also observed in *S. dysgalactiae* infections complicated by septic arthritis, ocular involvement, and vertebral osteomyelitis [11,12]. The patient’s prior episodes of bacteremia without an identifiable source support this hypothesis and highlight the organism’s capacity to seed deep musculoskeletal tissue.

Blood cultures remain central to the diagnosis of *S. dysgalactiae* infection and may provide the earliest indication of a deep-seated infectious focus. Group C/G streptococci are typically highly susceptible to β-lactam antibiotics, with penicillin as the treatment of choice and cephalosporins as reliable alternatives. True penicillin resistance is exceedingly rare; however, increasing resistance to macrolides and clindamycin, as well as occasional fluoroquinolone resistance, has been reported [14,15]. A transient rise in transaminases was noted during therapy, likely drug-related, which resolved on follow-up. Treatment duration is guided by disease severity, ranging from short courses for uncomplicated skin and soft-tissue infections to prolonged therapy for invasive or deep-seated infections such as endocarditis or osteomyelitis. In patients with severe β-lactam allergy, vancomycin may be used, although experience with alternatives such as linezolid and daptomycin remains limited [14,15].

In the present case, the isolate demonstrated preserved β-lactam susceptibility with clindamycin resistance. Antimicrobial therapy was individualized following infectious disease consultation and susceptibility testing. Although narrower-spectrum therapy would have been sufficient for a confirmed monomicrobial infection, amoxicillin-clavulanate was initially selected because of recurrent bacteremia and deep muscle involvement, in which an early polymicrobial soft-tissue infection could not be excluded. A prolonged six-week treatment course was chosen to reduce the risk of relapse associated with deep-seated infection. The transition from ceftriaxone to co-amoxiclav reflected a shift from bacteremia-directed therapy to broader soft-tissue coverage, with subsequent step-down to oral therapy after clinical stabilization.

This case underscores several important clinical lessons. First, *S. dysgalactiae* is an emerging invasive pathogen capable of causing deep musculoskeletal infection even in healthy adults. Second, the combination of focal musculoskeletal pain and bacteremia should prompt evaluation for myositis, particularly when symptoms involve atypical muscle groups. Third, MRI plays an essential role in early diagnosis, allowing timely exclusion of abscess or necrotizing disease. Finally, prompt initiation of β-lactam therapy is crucial to preventing progression to severe complications, including septic shock, multiorgan involvement, or necrotizing myositis.

## Conclusions

Infectious myositis caused by *Streptococcus dysgalactiae* is uncommon, particularly when it involves atypical muscle groups such as the sternocleidomastoid and pectoralis major in immunocompetent adults. Clinicians should maintain a high index of suspicion for deep muscle infection in patients presenting with focal musculoskeletal pain and concurrent group C/G streptococcal bacteremia. Early MRI is essential for confirming the diagnosis and excluding abscess or necrotizing disease. Prompt initiation of appropriate β-lactam therapy is associated with excellent clinical outcomes and is critical to preventing disease progression and serious complications.
